# How gender shapes practice choices among family medicine residents and early career family physicians: a Canadian qualitative study

**DOI:** 10.3399/BJGPO.2024.0158

**Published:** 2025-07-02

**Authors:** Anneka Sheppard, Amanda Gormley, Lauren Mills, Madeleine McKay, Fiona Bergin, Roetka Gradstein, Catherine Moravac, Ian Scott, M Ruth Lavergne

**Affiliations:** 1 Faculty of Medicine, Dalhousie University, Halifax, Canada; 2 Department of Health and Wellness, Government of Nova Scotia, Halifax, Canada; 3 Department of Family Medicine, Dalhousie University, Halifax, Canada; 4 Primary Care Research Unit, Department of Family Medicine, Dalhousie University, Halifax, Canada; 5 Department of Family Practice, University of British Columbia, Vancouver, Canada

**Keywords:** family medicine, qualitative research, social sciences, primary health care

## Abstract

**Background:**

The practice choices of family medicine residents and early career family physicians shape access to primary care. A growing proportion of family physicians are women.

**Aim:**

To examine how gender operates in shaping family physician practice choices and subsequent practice patterns.

**Design & setting:**

Qualitative interview data were analysed. Family medicine residents and early career family physicians from three Canadian provinces (Nova Scotia, Ontario, and British Columbia) participated in interviews.

**Method:**

Qualitative interview data were collected as part of a larger mixed-methods study. Eighty-eight interviews were transcribed verbatim and coded into several node reports, including one on gender. Reflexive thematic analysis was conducted to identify themes related to how gender impacts physician practice choices.

**Results:**

Many participants described multiple intersecting pathways through which it was apparent that gender shaped their career and practice choices. Others did not identify the impact of gender in this regard. Parenthood and caregiving were commonly discussed, as were clinical interests specific to women’s health; however, gendered expectations of patients and colleagues were also seen to shape choices. In this way, gender shaped choices directly, but also indirectly in response to gendered experiences and expectations.

**Conclusion:**

Findings support the need for structural reforms including: increased availability of collaborative team-based models, flexible work schedules, closure of gendered wage gaps, and integration of gender awareness training through academic and healthcare institutions. Consideration of how primary care policies differentially impact across clinician gender is key to future planning to support a changing workforce that meets patient needs.

## How this fits in

This study corroborates that clinical interests, expectations for parenting, and financial implications related to gender shape practice choice, as have already been identified in the literature. This analysis adds to these elements by identifying ways in which gendered experiences, including expectations of patients and colleagues, were also seen to impact practice choices. Findings support the need for structural reforms that mitigate the impacts of gendered patient expectations and the promotion and integration of gender awareness training in academic and healthcare institutions to address gendered expectations of colleagues.

## Introduction

A higher proportion of women are entering family medicine over time^1–4^ and it is widely recognised that practice patterns differ by sex and gender.^5–9^ Female physicians tend to have more patients who are female, and younger patient panels.^10^ Electronic health record data highlights that female primary care physicians experience increased workload^11,12^ and earn significantly less money than male physicians.^13–20^ In addition, being a woman has been shown to be negatively associated with access to academic, research, and leadership opportunities.^4,7,21–23^

It is widely recognised that choice of specialty is gendered, and there is considerable research on factors shaping choice of specialty (that is, family medicine and other medical and surgical specialties).^24–28^ However, less is known about how gender shapes practice choice within family medicine. For example, family physicians can choose to pursue a comprehensive generalist practice or a focused practice in an area such as care of older people, emergency medicine, sports medicine, or addiction medicine^29^ or they may have a mixed practice of comprehensive care with a focus. Family physicians may also choose to work in a practice alone, in a group physician practice, in an interprofessional team,^30,31^ or as a locum physician. Choice of payment models include: fee-for service, salary, or blended capitation. The ways in which residents and early career physicians approach these decisions and begin their practice can shape patients’ access to primary care. Therefore, the factors that affect these practice decisions are important to define.

The mechanisms through which gender shapes practice choices have not been explored in depth. Gendered biases are widely recognised within medicine^32^ but it is important to explore how these biases shape the practice choices of family physicians, and in turn the services they provide to patients. Given that gender is a socially constructed concept that creates expectations about how we interact with others, how we work in a professional setting, and how we work within family systems, it is important to understand how gender influences family practice choices.^33,34^ We know that gendered societal expectations put a higher burden on women to perform domestic and childcare work, which must be managed along with professional demands.^35–38^ Previous research has suggested that expectations from both patients^39–41^ and colleagues^7,42^ differ based on physician gender, but how this connects to practice choices has not been fully explored. One previous study has shown that family physician choices are influenced by a variety of factors including self-preservation within the current structure of the healthcare system, access to a support system, and training experiences through medical school and residency.^29^ It is expected that the experiences of these factors will differ by gender. In this analysis we examine how gender shapes practice choices for family medicine residents and early career family physicians.

## Method

### Study design and population

This study reports on a secondary analysis of a subset of qualitative interview data collected through a larger mixed-methods study. The larger study examined factors contributing to practice intentions and choices among resident and early career family physicians in British Columbia, Ontario, and Nova Scotia. In Canada family physicians complete 2-year residency training after 4 years of medical school. Early career is defined as up to 10 years from time of residency completion. The details of this study are available in the published protocol.^36^

Resident and early career family physicians were recruited for the qualitative arm of this larger study through family medicine residency programme email listservs in each province, Twitter (now known as X), Facebook, and Nova Scotia’s provincial medical association, Doctors Nova Scotia. Interested participants completed an online screening questionnaire that captured demographic information and practice characteristics (Supplementary Table S1). The questionnaire was developed and pilot-tested by the research team. Included options for gender were ’male’, ’female’, and ’prefer to describe’. That binary options and that male and female more commonly refer to biological sex is a limitation discussed below, but hereafter when referring to participant self-described gender, we use the language ’male’ and ’female’ to align with selected options, and ’man’ and ’woman’ to refer to genders more broadly. Purposive sampling was employed to maximise variation across participant characteristics in each province, including self-identified gender, marital status, dependants, training location, years of training, years in practice, scope of practice, and practice models.

Individuals selected through the above process were invited to participate in a 60-minute interview over the phone. Those who participated in an interview were provided with study information and an honorarium. Written consent was obtained. Of those selected, interviews were conducted with 31 out of 32 resident family physicians and 63 out of 69 early career family physicians. Reasons for non-participation included scheduling conflicts (*n* = 2), no response (*n* = 4), or withdrawal with no reason provided (*n* = 1). Interviews were conducted between April and October 2019. Participant characteristics are provided in Supplementary Table S1.

### Data collection

The interview guides included questions about the influence of gender on initial career plans (residents) and on one’s career (early career family physicians) among others. One research analyst per province (three total) conducted one-on-one interviews. Each research analyst was female, master’s level, and trained in qualitative interviewing. A semi-structured interview guide specific to each subgroup was used. Interviews were audio-recorded, professionally transcribed verbatim, and quality checked. Research analysts recorded their reflections and interview summaries after each interview.

### Data analysis

Iterative, inductive thematic analysis was conducted.^43^ For the qualitative arm of the larger mixed-methods study, three research analysts generated initial resident and early career family physician codebooks through inductive coding of one resident and one early career family physician interview. Codebooks were then refined through application to a subset of transcripts with guidance from the senior author. The research analysts used the final codebooks to code transcripts from their respective provinces in NVivo (version 12). Codebooks were iteratively amended to incorporate new codes and to ensure consistency between the resident and early career family physician codebooks. Interviews ceased when no new themes or sub-themes were identified by the research team.

The sample used in this article consisted of excerpts from interviews of 28 family medicine residents and 60 early career family physicians who spoke about the effect of gender on practice choices during their interview. This was determined by the research analysts who iteratively coded the transcripts into node reports including one gender node report for residents and one gender node report for early career physicians.

Eight of nine members of our research team identify as cisgender women; we share intersecting identities as clinicians, medical students, policy analysts, and researchers. All members were attentive to potential gender bias in this work. We routinely questioned the impact of our pre-conceived ideas about gender on our reaction to and interpretation of participants’ narratives. Data analysis for this manuscript was based on Braun and Clarke’s^43^ six phases of reflexive thematic analysis, rooted in social constructivism.^44^ This epistemological stance recognises that individuals create meaning based on their previous experiences combined with their interactions with others in their current context. A sense of knowledge or meaning is fluid over time as new experiences and social influences occur. In this work we learn about individual lived experiences, expecting that they will vary, and look for similarities that inform our understanding of the phenomenon we are exploring, with the ultimate goal of supporting actions that bring about equity.

The gender node reports were reviewed. Inductive coding was completed. Codes were revised and reorganised leading to potential overarching themes and sub-themes. This was done first using a spreadsheet with codes, descriptions of codes, general conclusions drawn from the codes, and example quotes. Miro, a mind-mapping platform (miro.com), was then used to draw connections between codes and to group them into overarching themes. A reflexive approach was used throughout. Themes were presented to members of the research team during the process for feedback on the analysis.

The trustworthiness of data analysis was ensured using multiple strategies including: (i) triangulating across a large sample size of participants with diverse experiences from three provinces and two participant groups;^45,46^ (ii) conducting data collection and analysis in interactive ways using multiple analysts;^47,48^ (iii) identification and reporting on outliers;^46^ and (iv) presenting to members of the research team who were not involved in the analytic process to confirm data interpretation.^46^

## Results

The 28 family medicine residents and 60 early career family physicians included in this article reported a variety of intended or current clinical areas of practice, encompassing both focused and comprehensive practice. Responses to a preliminary questionnaire found that 52 responders identified their gender as female, 35 identified their gender as male, and one preferred not to answer. Although gender is non-binary and fluid, and distinct from biological sex, our sample did not include any individuals who identified other genders.

While some participants did not directly identify gender as shaping their practice choices, many described multiple intersecting pathways through which it was apparent that gender shaped their career and practice choices. Parenthood and caregiving were commonly discussed, as were clinical interests specific to women’s health; however, gendered expectations of patients and colleagues also shaped choices. In this way, gender shaped choices both directly, as well as in response to gendered experiences and expectations. A number of responders noted the impact financial considerations had on their practice choices and the steps they took to mitigate the financial implications of the gendered expectations they felt. Our two major themes and sub-themes are outlined in Supplementary Figure S1 and discussed below.

### Theme: Some reflections on the influence of gendered experiences on practice choices were invisible or muted

#### Gender has no impact on career

When prompted about how gender has influenced practice choices or career plans, some participants did not feel that gender played a role. This response was more common among males than females, and among residents rather than practising physicians:

*‘That’s interesting. Probably not for me. No, I don’t think my gender has impacted my career plans. Well, I guess in a way it does because I get to choose what I want to do, and I just decide where I go. So probably in that way as a male, it helps. And it doesn’t limit me to as many things as maybe for the opposite sex.’* (Male resident, British Columbia [BC])

This pattern among male physicians initially expressing no sense of impact of gender on practice choices reflects their power position within social norms such that they do not perceive any restrictions on their choices thus highlighting the invisibly of gender on practice choices to some. Awareness of power differences can arise from experiences of discrimination and often, compromise. Without those experiences, the concept is not relatable or easily understood. It remains invisible. This quote references freedom, lack of limitation, and a partial recognition that people who are not male may not experience the same degree of freedom. See Supplementary Table S2 for additional quotes.

#### Factors other than gender drive practice choices

In a different way, some female participants did not feel that gender impacted their career planning. They spoke instead about preference for patient populations, or professional preferences that they did not identify as being driven by gender. Rather, the influence of personal values and role models were highlighted. In some cases, gendered experiences were shared that female residents and physicians claimed did not interfere with their commitment to their practice choices, which could be interpreted as a form of awareness and resistance. We have classified this type of response as ‘muted’. Collectively the interviews highlighted the complexity of the concept of gender and the multiplicity of interpretations held. It can be difficult to separate the influence of gender from personality traits, familial and societal norms, and the perception of free choice.

### Theme: Reflections about influence of gender on practice choices reveals multiple intersecting pathways

#### Gender is linked to clinical interests related to sexual and reproductive health care

Interest in services related to women’s health, or sexual and reproductive health care framed understanding of gender more broadly. Many women expressed interest in care that included obstetrics, gynaecology, maternity care, contraception counselling, intrauterine device (IUD) placements, and so on for a variety of reasons. For example, one participant described being interested in promoting women’s health because of a desire to help and empower women (female physician, Ontario [ON]). Another described being drawn to women’s health because she was more comfortable with a patient population reflecting her own gender (female physician, BC).

Some male participants described patient barriers as preventing them from including services for women’s health in their practices. They spoke about patient preferences for female providers. One male physician reported an interest in obstetrics who felt that gender-bias on the part of their female preceptor precluded adequate training opportunities (male physician, ON).

#### Reflections on gender were related to parenthood and specifically to gendered expectations of parenthood

Responders commonly discussed the impact that pregnancy and parenthood had on shaping practice choices. Female participants commented on the pressures they had experienced to reduce the length of their parental leaves, accommodations for breast-feeding while working, and greater responsibility overall compared with men for childcare responsibilities through the years. Female participants who expected they may become pregnant considered implications for coverage of their patient rosters; some made practice model decisions with future parenting in mind. Beyond pregnancy, participants reflected on gendered expectations for parenthood. Women reported experiencing judgment when they did leave their families to work the hours required of them, but also faced judgment if they prioritised their families by decreasing their practice hours.

Importantly, none of the male physicians discussed current or future parenting as having an impact on their careers. Only one male commented on parenting responsibilities:

*‘The obvious one is I don’t have to necessarily plan for any amount of maternity leave. I would like to take parental leave after I have kids. But I’m not kind of needing to. So I don’t necessarily have to plan for it, uh, and when. I guess that’s the other thing, is I have obviously less of a biological imperative to procreate before 35. … But I don’t know that it directly plays into my career plan per se*.’ (Male resident, Nova Scotia [NS])

Even in this example, while parental leave is mentioned, the broader role of parenting beyond parental leave was not expressed in the way it was by female participants.

Expectations for pregnancy and parenthood appeared to shape both the choice of family medicine as a career and attributes of family medicine practice. Family medicine was frequently described to and by participants as offering flexibility compatible with having a family. Considerations of stability of income as well as flexibility needed for parenthood were also reported as shaping practice choices within family medicine. Female participants noted that some aspects of practice such as hospitalist, emergency, or obstetrical work were weighed in the context of schedules and flexibility needed to fulfill family responsibilities. Many family physicians who were mothers reported being drawn towards a collaborative health clinic because it provided work–life balance. One female participant explained that many female physicians recommend waiting to buy into a practice until after they have had children (female physician, BC). Several female physicians said that they were also likely to wait until after their children were grown to pursue additional roles in leadership, education, or advocacy.

Some participants felt that it was parenthood rather than gender that most shaped choice, expressing that female physicians without children may make similar decisions to men; however, the intersecting influences of gender and parenting shaped choices.

#### Gendered patient expectations shape practice patterns and choices

Participants described how patients have different expectations of their physicians depending on the physician’s gender. Expectations are high for women to be more thorough, attentive, and empathetic making it harder for them to earn approval from their patients. These expectations lead to female physicians spending more time with each patient. This results in fewer patient visits and decreased remuneration, given that billing systems incentivise high volumes of patient visits. These heightened patient expectations of female physicians could cause them to seek out a practice with more team-based support or an alternate form of remuneration that doesn’t financially penalise them for longer visits.

While there was acknowledgement of higher patient expectations for female physicians, a few participants felt that it was within their capacity to manage this. For example, one resident explained:

*‘But again, I think there are probably some aspects of like being a woman that like I’m not really going to know the difference because I won’t ever have any other sort of experience. But I think just sort of like being cognizant of that. And I think I would definitely be like a more sort of like strict, I guess, physician. And that I wouldn’t just let patients ramble on, and I’d be on time, and things like that. Which I’ve seen like vary in practice by other people.* (Female resident, NS)

#### Practice choices are shaped by gendered expectations of colleagues and other healthcare providers

Female physicians also often struggled with heightened expectations from their fellow healthcare professionals, for example, being asked to take calls when not on call because she was known to be ‘*nice or kind*’ (Female physician, NS). Experiences and expectations from colleagues also resulted in female physicians describing having to work harder to earn the trust of other healthcare professionals, which included spending more time on tasks:

*‘Yeah, I think it’s just something that probably every young female doctor would tell you is something that impacts our practice. In terms of, you know, having to work a little bit extra hard to earn patient trust, having maybe to work a little harder to work with allied health in terms of needing their knowledge or help or kind of getting their trust maybe as well. And then the only other thing would be with patients and colleagues, and how you present yourself.’* (Female physician, BC)

Given these factors, some female physicians reported chosing practice environments specifically based on gender considerations:

*‘So I’m very fortunate that nearly every one of my workplaces is almost exclusively staffed by women and/or queer folk. So that’s been really nice for me. I haven’t run into as many of the sort of problematic kind of patriarchal hierarchies that definitely I saw a lot of in my training. So it’s definitely influenced the choices that I’ve made about where I want to work and who I want to work with.’* (Female physician, BC)

In addition to individual experiences of gendered professional expectations, the idea that the ’feminisation’ of family medicine is shaping the physician shortage was also raised by participants:

*‘You know, we’re the problem — that’s the attitude, is that we’re the problem. And like I heard that at a big meeting … an older physician came up to the microphone and basically said that, you know, we have an access problem because female physicians don’t want to work very much. And like nobody challenges it. Like people just let it go.’* (Female physician, BC)

This prevalent narrative continues to negatively impact female family physicians. Precisely how it shapes practice choices is currently unknown.

#### Gendered safety concerns may influence practice choices

Some spoke about personal safety concerns when considering practice locations in which they would be working alone, or in contexts in which patients could be demanding or threatening. One shared that she considered safety issues if, for example, she had just refused requests for opioid prescriptions to people who are ‘*rather aggressive*’; she would be worried about walking home from clinic:

*‘I think for me, like my weird dream is to be able to go to where drug users are using drugs and provide some sort of mobile health intervention … innovation. Of course my gender and my appearance is a factor, right, because security is always a concern. … Where if I was a big burly dude then I would be less afraid. But for me, I would have personal safety concerns for engaging in a project like that. Like I would certainly never do it alone. I don’t have that kind of bravery.’* (Female physician, ON)

Although some male residents and physicians may have similar concerns about working in specific contexts, safety issues were not raised by male participants in this set of interviews.

#### Gendered financial implications intersect with practice choices

Financial implications were evident in all themes. The impact of clinical interests, work time lost to parental leave, caregiving, and costs of childcare were all identified as having a financial impact. Patient expectations for longer appointments and professional expectations to receive lower compensation, or for some activities, no compensation, were more likely to be reported by females. Choice of alternate payment plans, or practice environments known to be non-hierarchical and gender inclusive were discussed in response to financial considerations.

## Discussion

### Summary

The participants in this study described several ways in which their gender impacts the choices they have made within their careers as family physicians. First, a passion for women’s health is a direct way in which a physician’s gender impacts their career interests and choices. On the other hand, physicians’ gender impacts their experiences in their career, shaping subsequent practice choices. These include patient expectations, professional expectations, gender-specific expectations around parental leave and parenting, and safety concerns in the workplace. Each of these are modifiable factors centred around gender, which is a concept created by society that is so deeply entrenched that it can be completely invisible. These modifiable factors are putting female physicians at a disadvantage in their personal and professional lives, ultimately affecting the sustainability of the healthcare system.

### Strengths and limitations

Our analysis adds to the existing literature through the identification of ways in which gendered experiences, including expectations of patients and colleagues, are seen to impact practice choices for female family physicians. The number of participants purposively selected to ensure diverse representation of demographic and practice characteristics demonstrates methodological rigour.

Participants only described their genders as male and female. We are not able to speak of the experiences of people with other genders, and this reinforces gender operating as a static binary rather than a fluid continuum.

Data collection for this study occurred before the COVID-19 pandemic and therefore may not be an accurate representation of today’s healthcare environment. However, literature since the onset of the COVID-19 pandemic has shown that female physician parents were even more impacted by adverse consequences of the pandemic such as work–family conflict, depressive symptoms, and isolation from working from home when compared with their male colleagues.^49^

Our study also only includes individuals who responded to requests to participate; however, we conducted purposeful sampling from a population that included broad representation from three provinces from across the country.

### Comparison with existing literature

Literature has shown that the landscape of family medicine in Canada is changing. While the number of family physicians in Canada is increasing, many Canadians are still facing challenges when accessing primary care. This comes alongside many primary care practices in Canada becoming less comprehensive and more focused, which could be contributing to patient challenges accessing comprehensive care.^10^ Another ongoing change being seen in primary care is the increase in women entering family medicine.^1–3^ This increase has resulted in women physicians being identified as the ’cause’ for current primary care access difficulties experienced by patients.^2,50^ Our work adds to the critique of these claims by exploring the socially constructed reasons why practice differs by gender.^1,39,40^

Documented practice differences between male and female physicians include female physicians working fewer hours per week, seeing fewer patients for longer visits, and providing more counselling and caring for more complex patients.^1–4,11,12,23,39,40^ This occurs within a broader social context where work that is feminised tends to be compensated less, which may apply to the counselling and administrative coordination that more complex patients need. Financial losses occur as a result of this and also related to time off for parental leave and parenting responsibilities, being paid less than their colleagues, and having fewer opportunities for leadership and academic roles.^4,7,12,13^ At the same time, female physicians also often require more flexibility in their practice in order to maintain work–life balance and tend to family or childcare needs.^20,36,37^ Our work corroborated these findings and has identified how these work and personal factors directly and indirectly influence female physicians’ practices. In Canada, tied to these circumstances, female physicians experience higher rates of burnout than their male counterparts.^51,52^ Importantly, when family physicians reduce their practice hours to mitigate burnout or leave the profession altogether, patient access to care is negatively impacted. Thoughtful educational and policy reforms are urgently needed.

The way gender impacts practice has been seen in other high-income settings with female Australian remote GPs being affected by gender roles imposed onto them by their colleagues.^38^ American physicians who are mothers also experienced gendered job expectations, financial inequalities, limited opportunities for advancement, lack of support during pregnancy, and barriers to work–life balance.^7,35–37^ Among female self-employed physicians in France, their gender pay gap was found to be attributed to the ’carer’ effect of childcare and family responsibilities after having children.^16^ Research out of Australia also showed that the ’carer’ effect of having children reduces working hours for females significantly more than for males.^38^ As family medicine becomes more feminised, and consequently devalued in the same way other feminised professions have,^49,53–56^ it is more important than ever to recognise and address how gender roles, gender expectations, and gender discrimination interact with practice choices. It is critical for practitioners, educators, funders, managers, and governments to address these realities to ensure a robust, equitable, and functioning healthcare system.

Our study corroborates that gendered differences in clinical interests, expectations for parenting, and physician compensation identified in the literature also shape practice choice. Similar experiences and influences were identified as contributing factors for both resident and early career family physicians demonstrating that these factors are consistent throughout the early years of a physician’s career. This analysis adds to these elements by identifying ways in which gendered experiences, including expectations of patients and colleagues, were also seen to impact practice choices for female family physicians.

### Implications for research and practice

This study highlights that many of the ways gender shapes practice are modifiable through structural reforms. Not only in Canada, but also around the world, widespread adoption of new practice models are needed, including collaborative team-based care featuring flexible work schedules. Payment models need to be implemented that recognise and remunerate the time spent providing comprehensive and empathetic care that patients expect, and payment models are needed to close gendered wage gaps.^4,13^ Given gendered colleague expectations and safety issues, we encourage the promotion and integration of gender sensitivity training in academic and healthcare institutions. Consideration of how primary care policies differentially impact across clinician gender is key to future planning to support a changing workforce that meets patient needs.

## Data

The datasets analysed within this manuscript are not publicly available due to participant confidentiality and research ethics board requirements.
